# Responding to measles outbreaks in underserved Roma and Romanian populations in England: the critical role of community understanding and engagement

**DOI:** 10.1017/S0950268820000874

**Published:** 2020-04-29

**Authors:** Sadie Bell, Vanessa Saliba, Gail Evans, Stephen Flanagan, Sam Ghebrehewet, Helen McAuslane, Bharat Sibal, Sandra Mounier-Jack

**Affiliations:** 1 Department of Global Health and Development, Faculty of Public Health and Policy, London School of Hygiene & Tropical Medicine, London, UK; 2 Department of Immunisation, Hepatitis and Blood Safety, Public Health England, London, UK; 3 Adults and Health Directorate, Leeds City Council, Leeds, UK; 4 Cheshire and Merseyside Health Protection Team, Public Health England, Liverpool, UK; 5 Yorkshire and the Humber Health Protection Team, Public Health England, Leeds, UK; 6 West Midlands East Health Protection Team, Public Health England, Birmingham, UK

**Keywords:** Health policy, measles, outbreaks, underserved communities, vaccination (immunisation)

## Abstract

Since 2016, the European Region has experienced large-scale measles outbreaks. Several measles outbreaks in England during 2017/18 specifically affected Romanian and Romanian Roma communities. In this qualitative interview study, we looked at the effectiveness of outbreak responses and efforts to promote vaccination uptake amongst these underserved communities in three English cities: Birmingham, Leeds and Liverpool. Semi-structured in-depth interviews were conducted with 33 providers involved in vaccination delivery and outbreak management in these cities. Interviews were analysed thematically and factors that influenced the effectiveness of responses were categorised into five themes: (1) the ability to identify the communities, (2) provider knowledge and understanding of the communities, (3) the co-ordination of response efforts and partnership working, (4) links to communities and approaches to community engagement and (5) resource constraints. We found that effective partnership working and community engagement were key to the prevention and management of vaccine-preventable disease outbreaks in the communities. Effective engagement was found to be compromised by cuts to public health spending and services for underserved communities. To increase uptake in under-vaccinated communities, local knowledge and engagement are vital to build trust and relationships. Local partners must work proactively to identify, understand and build connections with communities.

## Introduction

Measles is a highly infectious and potentially fatal viral infection which can be prevented by immunisation with the two-dose measles, mumps and rubella (MMR) vaccine [1]. Insufficient MMR vaccination coverage to provide herd immunity means that measles remains one of the leading causes of death globally in children. There were a staggering 110 000 measles-related deaths reported worldwide in 2017, mostly among young children [2].

Since 2016, the European Region has been affected by large-scale measles outbreaks. There were 83 540 measles cases and 74 measles-related deaths in the World Health Organisation (WHO) European Region in 2018, compared to 5273 measles cases and 13 measles-related deaths in 2016 [3]. The WHO European Region experienced 34 300 measles cases and 13 measles-related deaths in January and February 2019 alone [3].

England has also been affected by large measles outbreaks, and having achieved WHO measles elimination status in 2017, this was subsequently lost in 2019 due to an increase in the number of cases detected [4, 5]. Between 1 January 2018 and 31 December 2018, there were 966 laboratory-confirmed measles cases in England, increasing from 259 during 2017 [6].

Recent outbreaks in England have been linked to the travel of under-vaccinated people to measles endemic countries, and the subsequent transmission of measles to communities with poor vaccination coverage [7]. Several European outbreaks have been linked to travel within Eastern Europe, including to Romania [8–10], and found to disproportionately affect Roma communities [11–16]. Romania had an estimated coverage of 75% for two doses of MMR in 2017 and has experienced particularly large outbreaks in recent years [9, 17]. In 2017–18, several measles outbreaks occurred in England that specifically affected Romanian and Romanian Roma communities [7].

Roma are one of the largest and most marginalised ethnic minority groups in Europe and have experienced an extensive history of discrimination, persecution and social exclusion [17, 18]. In the UK, Roma experience inequalities in standards of living, education, employment and health [19–23]. Vaccination uptake has been reported as lower in Roma communities compared to non-Roma communities across Europe [24–26]. Roma communities in the UK have particularly been found to experience language and literacy, and discrimination, as barriers to vaccination and health service access [27].

There are few studies that have specifically looked at health service access amongst Romanian communities in the UK [28, 29]. Our previous research identified that Romanian communities may experience barriers to accessing vaccination and primary care services [28]. We found that community members experienced difficulties in navigating and trusting the National Health Service (NHS), overcoming language barriers and understanding differences in vaccination scheduling and delivery in the UK compared to Romania [28].

In this study, we aimed to explore the approaches taken by responders in the public health management of measles outbreaks in Birmingham, Leeds and Liverpool, three cities in which Romanian and Roma communities were particularly affected by measles outbreaks. This study was undertaken in parallel with research we conducted with Romanian and Roma community members in Birmingham, Leeds and Liverpool to explore factors affecting vaccination at the time of the 2017/18 measles outbreaks. We found that factors related to access and acceptance, such as language and literacy barriers, ease of registering with a general practice (GP) and trust in health services, were the main barriers to vaccination amongst the communities [30]. The largest contributors to under-vaccination in the communities were due to access barriers, linked to social determinants, rather than community members' actively refusing vaccination.

In this study, we investigated whether responders to measles outbreaks were able to overcome these barriers and considered the most effective ways of promoting vaccination uptake amongst underserved Romanian and Romanian Roma communities.

## Methods

Semi-structured in-depth qualitative interviews were conducted with providers involved in vaccination delivery and outbreak management in Birmingham, Leeds and Liverpool during the 2017–18 measles outbreaks. Our study focused on these cities given that the measles outbreaks in 2017/18 particularly affected Romanian and Roma communities in these areas.

### Recruitment and data collection

Eligible participants included frontline vaccinators (e.g. practice nurses and school nurses) and representatives from Public Health England (PHE) Health Protection Teams (HPTs), Screening and Immunisation Teams and Local Authorities. We identified and recruited participants working for PHE HPTs in each city, who then connected us to other providers they considered key in delivering the outbreak response. Further providers were identified using snowball sampling through subsequent participants.

The interviews lasted 30–45 min and took place in person or via telephone. All interviews were audio-recorded and conducted by SB. The interviews were conducted between June 2018 and January 2019.

Providers were asked about their experiences in delivering vaccination services to Romanian and Roma service users during the outbreaks, and the effectiveness of their approaches.

### Data analysis

Interviews were transcribed verbatim and analysed thematically using the stages outlined by Braun and Clarke [31]: data familiarisation, coding, and theme identification and refinement. To enhance the rigour of the analysis, coding approaches were discussed by the researchers. NVivo 11 was used to manage the data during analysis.

### Ethical approval

The study received ethical approval from the London School of Hygiene and Tropical Medicine Observational Research Ethics Committee (Ref: 12124), the Health Research Authority (Project ID: 224734) and from Research and Development departments in the recruitment areas.

## Results

Thirty-three providers took part in the study, an overview of participants is provided in Table 1. MMR vaccination coverage (Table 2), and the profile of measles outbreaks and their management differed in each city (Table 3).

**Table 1.** Participants

Participant's place of work	Brief description of role in outbreak response	Years working in the role	Number of interviews		
			Liverpool	Leeds	Birmingham
Public Health England (PHE) Health Protection Team (HPT) (e.g. Consultant in Health Protection, Nurse Consultant in Health Protection, Health Protection Practitioner)	– HPTs receive notifications of infectious diseases and are responsible for looking at the epidemiology of outbreaks – HPTs are responsible for convening and chairing outbreak control meetings/local incident management meetings/daily clinical review meetings – HPTs make risk assessments and use this, along with their insight into outbreak epidemiology, to inform disease prevention and control activities – Preventative and reactive role	3–5	1	1	5
Screening and Immunisation Team (SIT) (e.g. Screening and Immunisation Team Manager)	– Advise on the commissioning of vaccinations (under Section 7a of the Health and Social Care Act) – Offer vaccinations in schools and community settings		1	2	1

Practice nurse	Explaining, promoting and delivering vaccinations	2–25	–	4	1
General practitioner	Promoting GP access/vaccination uptake amongst underserved, including Romanian, communities	15	–	1	
GP practice manager	Involved in call/recall systems (inviting and reminding patients about vaccinations)	2–4	–	3	2
Local government (e.g. local authority public health team or local council divisions i.e. Education, Health)	Improving access to health services and vaccinations. Providing links to communities and helping to facilitate engagement	10–25	1	–	4
School Nursing Team	Explaining, promoting and delivering vaccinations		1	–	–
Community Immunisation Nursing Team	Working alongside GPs to improve vaccination uptake in underimmunised children in the community		2	–	–
Social Exclusion Team	Main role is supporting asylum seekers and w orking with Eastern European communities	1–10	2	–	–
Health visitor	Explaining and promoting vaccinations		–	–	1
**Total = 33**			**8**	**11**	**14**

**Table 2.** MMR vaccination coverage for England and the cities of Birmingham, Leeds and Liverpool (2017–18)

	MMR 1^st^ dose at 24 months (2017–18)	MMR 1^st^ dose at 5 years (2017–18)	MMR 1^st^ and 2^nd^ dose at 5 years (2017–18)
Birmingham	87.6%	93.7%	81.6%
Leeds	92.9%	95.8%	88.2%
Liverpool	93.0%	95.0%	86.2%
**England**	**91.2%**	**94.9%**	**87.2%**

**Table 3.** Outbreak responses in Birmingham, Leeds and Liverpool

City	Outbreak -first cases	Outbreak size	Outbreak response approaches
Birmingham	November 2017–May 2018 (outbreak confirmed closed end June 2018). First cases reported in Romanian and Romanian Roma communities. Cases presented to A&E and primary care settings	116 confirmed cases. 20 probable cases	– Door-to-door vaccination (health visitors and screening and immunisation team going to the homes of known cases and their contacts) – Media communication – GP incentivisation scheme – Extra GP sessions – School-based immunisation (focusing on schools in the outbreak areas with lowest MMR update) – Heightening awareness of measles/measles management in healthcare services
Leeds	First cases reported in Romanian and RomanianRoma communities. First cases linked to a primary school, and wider spread linked to exposure in hospital settings	36 confirmed cases. In total, around150 confirmed, probable and possible cases	– Vaccination in a primary and secondary school (within a week of first disease notifications) – GPs inviting/reminding parents/guardians about missed vaccinations – GPs organised an extra clinic in one of most affected postcode areas – Door-knocking approaches, trying to encourage registration at general practice – Vaccination through community centre linked to a school – Training of front-line staff working with the communities – Media communication
Liverpool	First cases reported in Romanian and Romanian Roma communities	22 confirmed cases	– Mobile vaccination vans (supported by a Roma community worker) – Offer of meal and vaccination in community centre (facilitated by the community centre manager)

The factors that influenced the effectiveness of responses in reaching underserved Romanian and Roma communities were categorised into five themes. These themes were: (1) the ability to identify the communities, (2) provider knowledge and understanding of the communities, (3) the co-ordination of response efforts and partnership working, (4) links to communities and approaches to community engagement and (5) resource constraints.

### Identifying underserved communities

It was essential for providers to be able to correctly identify the communities affected by the outbreaks in order to optimise their responses. With vaccination coverage data only available at local authority, clinical commissioning group (CCG) and GP level, it was challenging for providers to identify ‘pockets’ of lower uptake within the population, leaving undervaccinated communities at risk of vaccine-preventable diseases.

‘…. every year we drill down to practice level but unless you know where a specific community sits then it can be difficult to say, ‘Oh yeah, that's that community.’’ (Provider#25)

To identify under-vaccinated communities required providers to ‘*pull different data sources together*’, including school-level data, which provided student nationality and country of birth data.

Healthcare professionals routinely record childhood vaccination uptake data in GP records and through the child health information system. Providers found that communities affected during the outbreaks were not necessarily registered with a GP or attending school, and therefore they were not captured in GP or school-level data. Instead, engagement with other sources of information was essential to identity communities at risk of under-vaccination. Leeds, for example, used National Insurance number registrations to identify recent migration trends.

‘We did a lot of mapping work around vaccination and National Insurance registrations which just helped to target our resources. Very unsurprisingly vaccine rates are low where there was a lot of National Insurance number registrations anyway. It helped focus the attention I think on what … where we needed to put our energies.’ (Provider#1)

Several providers reported being aware of the existence of unmet health needs amongst Romanian and Roma communities ahead of the outbreaks but felt unlistened to and hence restricted in their abilities to address inequalities within the communities. It was perceived that the communities only came to public health attention with the outbreaks.

### Knowledge and understanding of the communities

Within each city, several providers reported that as the outbreaks unfolded, they knew very little about the Roma and Romanian communities affected. In many instances, providers had not made a distinction between Roma and Romanian and the terms were sometimes used interchangeably.

‘[There was] so much confusion at the start…about whether we were talking about Roma communities or Romanian nationals. They are clearly not the same groups. They have very distinct cultural behaviours. Language issues. Religious influence. Cultural behaviours; and that all drives their engagement in the healthcare system…. there are [also] sub-groups within the populations.’ (Provider#11)

At the start of the outbreaks, providers believed that the preferred language of community members would be Romanian. In some instances, this was this case, but many Romanian Roma do not speak Romanian as their first language (instead speaking Romani, which has many different dialects) and literacy may also be a barrier.

Views from several providers inferred negative attitudes towards the communities. Some interviewees did not comprehend why community members may be distrustful of healthcare professionals and have different beliefs around health. Without having context to the history of discrimination and persecution towards Roma, healthcare professionals instead became frustrated in trying to reach the communities.

Poor understanding of communities and factors affecting vaccination impeded effective strategies to manage the outbreaks. Understanding the communities was crucial to effective outbreak management.

‘It made a big difference to who was round the table because it meant then that when I started phoning around to get people to come to the outbreak meeting, we got the right people. So, we got the manager of the Children's Centre where the Roma population [go]. We got one of the practice managers from one of the local practices. We got somebody from the local school that's 65% Roma, so we got the right people there.’ (Provider#28)

### Co-ordinating efforts and partnership working

In some instances, providers reported that there were challenges in identifying responder roles and responsibilities and promptly co-ordinating efforts.

‘Perhaps we need to think about [an integrated] model of working with our local authority partners…. Rather than, “That's your job. That's your job,” Where there's overlap no one's really doing it, no one's coordinating it, and then it becomes very much, “You didn't do this. Why didn't you do that?” rather than, “How can we make this happen?”’ (Provider#16)

In a few instances, communication difficulties were reported between PHE teams and frontline healthcare professionals. PHE teams had little power to enforce some of the recommendations as part of the outbreak (e.g. such as requesting busy GPs to support contact tracing for patients sitting in the surgery). In one city, PHE communication with hospitals was difficult due to large numbers of cases being reported, admitted and requiring extensive contact tracing.

‘….in hospitals there were issues with communication, certainly, because they weren't notifying us. We ended up with a system where we would contact them every day to ask them, “Do you have anybody who's been tested for measles?” Whereas, it should have been the other way around.’ (Provider#16)

Trying to mobilise responses, considering competing pressures on services, was also problematic.

‘…. Who's paying for it all? That was our main beef with the GPs. We, as an organisation, would tell them, “We need this Public Health action to happen,” but the GPs would come back and say, “I haven't got the time, I haven't got the staff or resources, …”’ (Provider#15)

In one city, efforts between partners were easier to define and co-ordinate the following lesson learnt in a past outbreak, in the same population and postcode area.

‘….we were lucky in Leeds actually because we'd done a lot of work last year around roles and responsibilities in response to an outbreak, so actually we had a lot of shared understanding about right, well the CCG's role is this, the PHE's role is this, the local authority role is this. It made life a lot easier’ (Provider#1)

### Provider approaches to community engagement

To prevent further outbreak spread, HPTs worked to trace the contacts of measles cases. HPTs in each city communicated with cases over the phone to ascertain the number of contacts, number of children in the household, travel history and length of stay in the country. However, interviewees reported that getting this information was problematic, particularly over the phone, given language barriers and a lack of community member trust in healthcare providers.

Though two of the cities reported good links with communities, providers in one city noted some difficulties. In addition, the lack of formal outreach mechanisms impeded the response.

‘We had minimal [engagement] because the outbreak started around November and as coincidence would have it, the Roma outreach support service that the local authority commissioned from the Red Cross; that ceased in November. I don't think it was a link, but it was just unfortunate timing, so it meant we had no real outreach apart from the [City Council] education team outreach that they do.’ (Provider#11)

Providers reported outreach approaches, such as door-to-door knocking, using mobile vaccination vans and offering vaccinations in community centres, as effective in increasing vaccination uptake. Key to the effectiveness of out-reach strategies was being able to take advantage of pre-existing links and established trust with the communities.

Vaccination in the community centre in Liverpool was particularly effective as the centre was considered ‘*part of the community not the establishment*’.

‘we put on a community meal in the evening in the Children's Centre. Now [the Children's Centre manager] does that regularly so she feeds these kids in school holidays because if they don't get a school dinner, they go short. So that's not unusual…. We just blitzed the area with flyers, that our Romanian Consultant translated… we immunised 80 children on the Monday night in about two-and-a-half hours.’ (Provider#28)

Other approaches used by providers, which may well have worked with other communities, such as putting on additional clinics in GP and incentivising GPs to vaccinate, were less successful. This was largely linked to the lack of community engagement with GP.

In Birmingham, providers had looked to outbreak responses used in Liverpool and Leeds but felt that vaccination through children's centres would not work for their communities, as Romanian and Roma families were reportedly not accessing them.

It was evident, in speaking with frontline healthcare providers, that in each city there were real attempts being made to engage with Romanian communities, e.g. employing Romanian speaking receptionists, sending letters and SMS messages in Romanian, holding additional clinics and trying outreach approaches. Providers in the three cities highlighted that encouraging communities to access GP was particularly difficult, and that the system of booking future appointments was often ineffective.

‘…. The biggest problem we think exist is if you send them pre-booked appointments, so if you get them to book an appointment that just does not work; they don't live like that. …. even if they've booked them themselves, it doesn't work, they did not attend (DNA) rates are very high.’ (Provider#12)

Some providers discussed using points of engagement with community members for vaccination to also promote registration with GP. This approach was more straightforward in Liverpool when the centre used for vaccination was within easy proximity of a GP.

‘…. The evening we did the meal and immunising, we were also literally taking people by the hand to register at the GP practice while we had them.’ (Provider#28)

Overall, the most effective approaches to outbreak management were multi-faceted and relied on insight into the communities.

‘…. that triple-pronged approach, so doing the schools, the GP practices, and the community at the same time helped sort of bring it all together in some way so you're targeting different parts of the population.’ (Provider#1)

### Resource constraints

#### Communication services

Face-to-face communication was seen as key to successful engagement. However, participants reported difficulties in sourcing and funding translators and interpreters. Finding Romani speakers was nearly impossible, given the absence of professional Romani interpreters, and prompt access to Romanian speakers could prove difficult. In one of the cities, leaflets had been developed in Romanian but the interpreters stated that the wrong dialect was used in these.

In community and GP settings, several participants discussed using hand gestures to communicate when they ‘*don't have time to get an interpreter*’, or using Google translate during consultations. This raised concerns that messages would be lost in translation, even with the use of telephone interpreters through LanguageLine.

‘…. You sometimes wonder what the Language Line is actually saying, because you're trusting their interpretation. Sometimes the patient looks totally confused with the Language Line.’ (Provider#14)

In several instances, participants had appealed to the help of charitable and community organisations and relied on their goodwill to provide translators and interpreters, or ‘fallen lucky’ in having Romanian speakers within their teams.

Participants working in PHE HPTs also struggled with language barriers, particularly as their work was reliant on over-the-phone communication with people diagnosed with measles and they needed to ask personal questions.

#### Perceived ‘unfairness’ of immunisation target payments

GPs receive financial payments for administering childhood vaccinations, based on achievement against a 70% or 90% uptake target for children at 2 and 5 years. Practices achieving the 90% target secure the highest financial payment. GPs that served populations with greater barriers to health service found it difficult to achieve the higher immunisation target.

‘…. The system is so biased in or towards practices in nice leafy-green areas with English [speaking] people because, you know, that our nice or well-off end but we hit 90 percent vaccination with no trouble at all. We don't have to do anything. Whereas there we spend a huge amount of effort and money and time and we hit about 77 percent.’ (Provider#12)

Trying to improve vaccination uptake with underserved communities was considered resource intensive (e.g. there may be a need for outreach approaches, translation of documents). The focus of the immunisation target payment system on outcome, rather than process, meant that providers felt penalised for not reaching targets even when they ‘*work so hard for immunisations*’. Providers also felt that this could lead to reduced vaccination call-recall efforts.

#### Cuts to services

Several participants reported cuts to community link services (e.g. community link workers, health visiting teams) as a major factor affecting the ability to engage with the communities during the outbreak. This was considered short-sighted and not cost-effective in the long run.

‘…a few years back [we] had community link workers and their job was to engage with communities and provide them with help and support so that trust develops. So, over the years when the economic crunch happened, they were the first ones, unfortunately, to get their jobs cut. So, when the outbreak happened there were no link workers as such. So, we were stuck, because the local authority had no contact, or very minimal contact with these communities’ (Provider#17)

With the cuts, there were frustrations about lost opportunities to discuss and administer vaccinations.

‘….we used to run our baby clinic and we used to have health visitors or school nurses in alongside us and that worked well and we got people who just came for weighing who we could then immunise. But they've since moved all of those people out, they say they've not got enough resource to have these people in here. So yet again….the changes and the cuts in the system are actually having the impact’ (Provider#4)

## Discussion

We found that improving MMR vaccination uptake and effectively managing measles outbreaks relied on (1) being able to identify and understand undervaccinated communities, (2) having links with undervaccinated communities and using appropriate approaches for community engagement, and (3) working with local partners to co-ordinate efforts. These three factors were found in turn to be largely dependent on available funding and resources, and efficient and creative partnership working. The research has highlighted the importance of having clear financing and working arrangements in place to respond to outbreaks.

One of the first challenges that providers faced was being able to identify under-vaccinated communities, particularly those not accessing GP or the school system. In 2017/18, 48.5% of Gypsy/Roma children in England were persistently absent from school [32]. The only ethnic group with higher persistent school absenteeism in 2017/18, at 65.2%, were Travellers of Irish Heritage [32]. Poor uptake of primary healthcare registration has been indicated in other populations, including certain migrant groups [33], and is a recognised risk factor for sub-optimal vaccination.

One resource that could be used by providers to bring a range of existing datasets together to identify under-vaccinated communities is the Strategic Health Asset Planning and Evaluation (SHAPE) Atlas [34]. SHAPE allows users to visualise layered maps of multiple datasets (e.g. location of healthcare facilities) and indicators (e.g. deprivation, ethnicity, age profile) for their local area. Users can also add further content to SHAPE that is relevant to their role, e.g. vaccination coverage data.

Once at-risk communities are identified, it is important that providers use appropriate engagement approaches to promote vaccination [35]. Knowing and understanding communities is paramount to this, and is exemplified in research with other communities with sub-optimal vaccination coverage, including the Charedi community in London [36]. Although there is a lack of research on the effectiveness of approaches to improve Roma access to and engagement with services [37], approaches that worked best in reaching the Romanian and Roma communities in the outbreaks were multi-faceted, involving GP practices, schools and the communities. Having strong links or contacts working directly with local communities (e.g. children's centre managers) was reported as essential and providers should work with community members to develop trust and explore factors affecting vaccination. Traditional approaches recommended to improve access to vaccination in primary care, such as sending invitations and reminders [38], may not be effective or may require adaptation to reach underserved communities. Other resources have highlighted the importance of working with children's centres and other community settings to reach under-vaccinated communities [36, 39].

Outreach approaches appeared to be needed, at least in the short-term, to develop trust and links with communities which can be used to promote access to GP and to other vaccinations. It was noted by providers that although their efforts during the measles outbreaks were focused on improving MMR vaccination rates, there were also concerns about sub-optimal coverage for other vaccinations.

We found that providers were severely limited by resource-constraints, including access to translators and interpreters, community link workers and community organisations. Resource constraints can be linked to changes introduced under the Health and Social Care Act 2012 when public health responsibilities shifted from national to local government [40]. Public health services are inadequately funded, with a £700 million cut in real term funding between 2014/15 and 2019/20. This has resulted in large cuts to community services, including school nursing and health visiting servicesf [41, 42], and the decommissioning of district immunisation coordinators [43]. The lack of a dedicated immunisation coordinator in local areas means that there is limited oversight of the immunisation programme in the area, and reduced scrutiny of immunisation coverage in underserved communities. Services for ethnic minority and Roma communities have also been specifically affected by spending cuts [44].

In general practice, providers felt that their ability to engage with and improve uptake in underserved communities were exacerbated by an immunisation target payment system focused solely on outcomes and not process. Immunisation target payments for GP are currently under review by NHS England, with a focus on trying to reduce inequalities in vaccination [45, 46]. Over the next 2 years, with changes to the GP contract, a new incentive payment system for immunisation will be introduced [47].

Challenges were voiced by general practice providers in tailoring culturally appropriate approaches to an increasingly ethnic diverse population. One recommendation that emerged from the interviews was to try and share more resources, such as translators and interpreters. It was also highlighted that more opportunistic vaccination or reminding about vaccination should take place, e.g. flexibility in giving vaccinations when a child presents in general practice for another reason.

Cuts to health visiting and school nursing teams also appear to have had an impact on maintaining links with underserved communities and the capacity to promote access to GP and vaccination. Short-sighted cuts to services have negative economic, societal impacts and public health impacts [48], and also affect other services including access to education and social support. There needs to be a concentrated effort, not just on vaccinations, but to encourage engagement with health services more broadly.

There is a limited evidence base on interventions to improve vaccination amongst potentially underserved groups, including Roma [37] and migrant communities [49], and an urgent need to formally evaluate approaches being used.

The measles outbreaks in 2017/18 served as a wake-up call to reach undervaccinated communities. The GP contract now includes a check of MMR status for 10- and 11-year olds [50], and lessons learnt from the measles outbreaks have been adapted into a PHE resource for local government [39]. The PHE resource – *Making measles history together* – contains a set of prompts to action focused on reaching undervaccinated communities by drawing on existing third sector and local authority resources [39]. The resource also contains a measles poster and communications leaflet available in English and Romanian [39]. Developing and building trust with communities so that they access services will not happen overnight. It will take time, and it is important that providers do not under-estimate the importance of small improvements in uptake in moving towards community protection.

Findings from our study may be applicable to other underserved communities, particularly those not accessing general practiceor the school system, who may be unfamiliar with vaccination delivery in the UK and experience language and literacy barriers.

## Strengths and limitations

The quality of the study was enhanced through the use of interviews with providers across three different outbreak settings to corroborate findings and develop a greater understanding of effective approaches to identifying and engaging with underserved communities. However, there were some limitations. Although we recruited a range of providers in each city, the range varied between cities. The study also did not focus on how the outbreak responses were funded. Future research should look at which partners fund activities and how funding decisions are made.

Interviews for this study were limited to providers; however, in preceding studies, we have included Romanian and Roma community members to explore factors affecting vaccination uptake [27, 30].

## Conclusion

Our study identified partnership working and community engagement as key to the prevention and management of the 2017/18 measles outbreaks that specifically affected Romanian and Roma communities. Without prior understanding and linkage with Romanian and Roma communities, it was difficult for providers to identify and access communities, who may not trust providers. Cuts to public health spending and the provision of services for underserved communities, including under-vaccinated Romanian and Roma communities, have far-reaching implications for reducing inequalities in vaccination.

To increase uptake in under-vaccinated communities, such as the Romanian and Roma communities in this research, local knowledge and engagement are vital to build trust and relationships. Local partners must work proactively to identify, understand and build connections with communities. Approaches that appeared to work best in reaching the Romanian and Roma communities in the outbreaks were multi-faceted, involving GP practices, schools and most importantly community representatives.
